# Cheese as an Article of Diet

**Published:** 1891-10

**Authors:** J. H. Kellogg

**Affiliations:** Battle Creek Sanitarium


### CHEESE AS AN ARTICLE OF DIET.

[Extract from lecture by Dr. J. H. Kellogg, Battle Creek Sanitarium.]

In the digestion of food, particularly of flesh foods and cheese, poisonous ptomaines are formed, but usually one does not find these in great quantities, from the fact that the liver is able to dispose of the most of them. The small amount of cheese ordinarily taken at a meal will not likely have any appreciable effect. But suppose a person takes a pound of cheese, then he will get enough poison for toxic effects. Two of the worst cases of poisoning I ever knew were from eating cheese. One of the patients died. Cheese always contains these ptomaines. The process of ripening is always a process of decay, and when taken into the stomach the liver has extra work to do to dispose of these poisons. The old saying, "Cheese, thou mighty elf, which digests all things but thyself," is not true. Cheese does not help digest other foods. I met a doctor who explained why it was that cheese was useful as an article of diet by saying that cheese contains gastric juice from the calf's stomach used as rennet. I asked him to explain why it was that new cheese does not have as much of this peculiar strong acid flavor as old cheese, and whether the rennet had the capability within itself of multiplying. This burning, fiery-tasting fluid which comes up in the throat and which the old doctor thought was gastric juice, is really butyric acid. It results from the decay of fats and oils and belongs to the category of ptomaines.

Sometimes cheese contains ptomaines of a kind particularly deadly, and they are always present. Professor Vaughn, of the Michigan University, discovered this poison in cheese and named it tyrotoxicon. He discovered also a very easy test by which its presence could be detected, and thought in the interest of the State board of health that it would be a good plan to supply all grocers with it. But first, in order to test it still further he collected a hundred specimens of different cheeses, and to his surprise every single one showed the presence of tyrotoxicon, so he did nothing more about submitting his tests to the grocers, for if followed out it would destroy the whole cheese business. When Professor Vaughn wants specimens of tyrotoxicon, he gets a quantity of cheese, pours a little milk and water on it, puts it in a bottle, and in due time he has plenty of the poison. If we must eat cheese it can be rendered much less harmful by being cooked. But one who wants to keep his liver in good condition and have a good healthy circulation, should avoid food which in itself is full of germs.

